# J-shaped association between body roundness index and depression: A large-scale cross-sectional study

**DOI:** 10.1097/MD.0000000000048718

**Published:** 2026-05-22

**Authors:** Jian Li, Lichun Zhou

**Affiliations:** a Department of Neurology, Beijing Chao-Yang Hospital, Capital University of Medical Sciences, Beijing, China.

**Keywords:** BRI, cross-sectional study, depression, NHANES, threshold effect

## Abstract

Depression is often linked to obesity. The body roundness index (BRI) offers a nuanced assessment of body fat distribution. This study investigates the BRI-depression relationship using NHANES data (2005–2018). We conducted a cross-sectional analysis of NHANES adult participants. Depression was assessed using a standardized scoring system. Multivariable logistic regression adjusted for demographic, lifestyle, and health-related confounders. Smooth curve fitting and threshold effect analysis were performed. A J-shaped relationship was identified between BRI and depression. Below BRI 3.21, each unit increase associated with 19% reduced depression risk (OR = 0.81, 95% CI: 0.69–0.96, *P* = .0127). Above BRI 3.21, each unit increase associated with 6% greater depression risk (OR = 1.06, 95% CI: 1.04–1.08, *P* < .0001). Both low and high BRI values link to increased depression risk, emphasizing the importance of maintaining optimal BRI for mental health.

## 1. Introduction

Depression is a prevalent mental disorder characterized by persistent low mood, loss of interest, and reduced energy, severely impacting an individual’s quality of life and posing a significant burden on society.^[1-3]^ According to the World Health Organization, depression affects approximately 100 million people worldwide and is one of the leading causes of disability.^[4]^ The pathogenesis of depression is complex and multifactorial, involving genetic, neurobiological, and environmental factors. Recent studies have suggested a potential link between obesity and depression.^[5,6]^ Obesity, particularly central obesity, has been associated with various metabolic and psychological disorders, including depression.^[7,8]^

Traditional measures of obesity, such as body mass index (BMI) and waist circumference, have limitations in accurately assessing body fat distribution and its associated health risks.^[9,10]^ The body roundness index (BRI) is a novel measure that considers both height and waist circumference, providing a more accurate assessment of body fat distribution and visceral adiposity.^[11,12]^ Previous studies have shown that BRI is a strong predictor of cardiovascular disease, diabetes mellitus, and other metabolic disorders.^[13,14]^ However, the relationship between BRI and depression remains unclear.^[15]^ The National Health and Nutrition Examination Survey (NHANES) is a comprehensive database that provides nationally representative data on the health and nutritional status of the US population. This study aims to investigate the association between BRI and the risk of depression using data from NHANES 2005–2018.

## 2. Materials and methods

### 2.1. Study population

**Figure 1.**
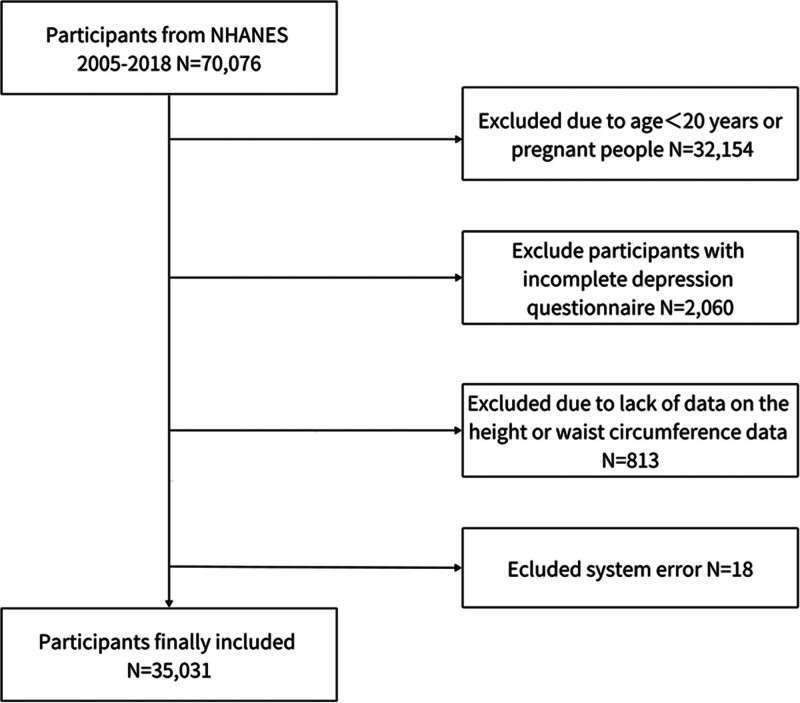
Flowchart of the sample selection from NHANES 2005–2018. Initially N = 70,076; exclusions: age < 20 years or pregnancy (N = 32,154); incomplete depression questionnaire (N = 2060); missing height or waist circumference data (N = 813). Final analytic sample: N = 35,031. NHANES = National Health and Nutrition Examination Survey.

This cross-sectional study utilized data from the NHANES conducted between 2005 and 2018, a comprehensive, nationally representative health assessment program. NHANES employs a sophisticated multistage probability sampling design to ensure representative sampling of the noninstitutionalized US civilian population. The survey methodology integrates detailed in-home interviews and standardized physical examinations performed at mobile examination centers, capturing comprehensive health and nutritional data. Participants undergo extensive assessments, including medical history interviews, physical measurements, and laboratory tests conducted by trained healthcare professionals. The collected data are rigorously de-identified and publicly accessible through the Centers for Disease Control and Prevention (CDC) website (http://wwwn.cdc.gov/nchs/nhanes/default.aspx). A flowchart of the sample selection from NHANES 2005–2018 is shown in Figure 1.

The study population comprised 35,031 adults aged ≥ 20 years, selected using a stratified, multistage probability sampling approach. Inclusion criteria were: adults aged ≥ 20 years and complete depression screening and anthropometric data. Exclusion criteria included: incomplete Patient Health Questionnaire-9 (PHQ-9) depression screening, missing critical anthropometric measurements (height and waist circumference), and system error N = 18.

The NHANES study protocol was approved by the National Center for Health Statistics Ethics Review Board, and all participants provided written informed consent prior to participation, ensuring rigorous ethical standards and participant protection.

### 2.2. Primary variables

The primary exposure variable was the BRI, calculated using the formula:


$$\begin{array}{l} BRI=364.2-365.5\times \sqrt{1-\frac{{\left(waist\;\;\;circumference\left(cm\right)/2\pi \right)}^{2}}{{\left(height\left(cm\right)/2\right)}^{2}}}. \end{array}$$

BRI was analyzed both as a continuous and categorical variable (tertiles), providing a comprehensive assessment of body fat distribution.

### 2.3. Outcome variable

#### 2.3.1. Depression assessment

The primary outcome variable was depression status, operationalized using the PHQ-9, a validated and widely used screening instrument for depressive symptoms. Each item on the PHQ-9 is scored from 0 to 3, representing the frequency of symptoms, with total scores ranging from 0 to 27. A score of ≥10 was defined as clinically significant depressive symptoms, a threshold consistently used in epidemiological research and validated through extensive clinical assessments.

#### 2.3.2. Covariates

Covariates were selected based on known confounders from previous literature and clinical practice. These covariates encompassed demographic, socioeconomic, lifestyle, and clinical domains. The demographic characteristics included gender, age (analyzed as a continuous variable), and race/ethnicity, which was stratified into Mexican American, other Hispanic, non-Hispanic, and other categories. Socioeconomic indicators included education level (categorized as less than high school, high school, and higher than high school), marital status, and poverty–income ratio (PIR), divided into 3 groups (<1.30, 1.31–3.50, and >3.50). Lifestyle factors were assessed through evaluations of smoking status (never smoked < 100 cigarettes, former smoker, and current smoker) and alcohol consumption (never drank < 12 drinks lifetime and drinkers ≥ 12 drinks annually). PA data, obtained via self-reported questionnaires, were stratified into 3 groups: active (≥ recommended activity level), less active (< recommended activity level), and inactive (no activity), aligned with the recommended 75 minutes of vigorous or 150 minutes of moderate PA per week. The clinical characteristics features include chronic disease assessment. Hypertension is defined as: mean systolic blood pressure ≥ 140 mm Hg and/or diastolic blood pressure ≥ 90 mm Hg, diagnosed by a doctor, or the use of blood pressure medications. Diabetes is identified by self-reported physician diagnosis, fasting blood glucose ≥ 7.0 mmol/L, or glycosylated hemoglobin ≥ 6.5%. Cardiovascular and cerebrovascular diseases are characterized by self-reported diagnoses of at least 1 condition: stroke attack, coronary heart disease, congestive heart failure, angina or heart attack.

### 2.4. Statistical analysis

Continuous variables were described as means ± standard deviation (SD) or median with interquartile ranges (IQR). Categorical data were presented as numbers and percentages. We employed generalized additive models (GAM) to explore the potential nonlinear relationship between the BRI and depression risk. Logistic regression models were used to estimate the association between BRI and depression, with results expressed as odds ratios (ORs) and 95% confidence intervals (95% CIs). After comprehensive consideration, we adjusted for the following covariates: age, sex, race/ethnicity, education level, marital status, poverty–income ratio, smoking status, alcohol consumption, PA, hypertension, diabetes, cardiovascular diseases, and hyperlipidemia.

In order to examine potential threshold effects, we utilized a 2-piece-wise linear regression model to identify possible nonlinear associations between BRI and depression risk. The turning point was determined through exploratory analyses, systematically moving the trial point along a predefined interval and selecting the point yielding maximum model likelihood. We conducted log-likelihood ratio tests to compare 1-line linear and 2-piece-wise linear models.

Dummy variables were used to handle missing covariate values. Statistical significance was set at a 2-sided alpha level of 0.05. All statistical analyses were conducted using EmpowerStats (www.empowerstats.com) and R software (version 3.6.1, http://www.r-project.org).

### 2.5. Ethical considerations

This study utilized publicly available, de-identified NHANES data, which received prior approval from the National Center for Health Statistics Research Ethics Review Board. As the data were anonymized, formal informed consent was not required. All analyses adhered to the ethical guidelines outlined in the Declaration of Helsinki and maintained strict data confidentiality protocols.

## 3. Results

### 3.1. Baseline characteristics

In this comprehensive cross-sectional study involving 35,031 participants, we systematically unveil the intricate associations between BRI and demographic characteristics, lifestyle factors, and health status through tertile stratification. Demographic analysis revealed significant shifts in population age structure as BRI gradients increased: the first BRI tertile predominantly comprised young adults aged 20 to 40 (58.29%), while the third tertile was characterized by individuals over 60 (38.48%). Gender distribution demonstrated notable changes, with males dominating the low BRI tertile (53.68%) and females significantly increasing in the high BRI tertile (60.35%). Lifestyle patterns showed increased current smoking rates (27.52% vs 15.72%) and proportions of nondrinkers in the high BRI tertile. Clinical indicators revealed substantially elevated rates of diabetes (28.72% vs 4.59%), hypertension (50.30% vs 16.32%), and cardiovascular diseases (14.70% vs 4.64%) in the high BRI tertile. Notably, depressive symptom detection rates incrementally increased from 6.67% in the first tertile to 11.71% in the third tertile (Table 1).

**Table 1:** Demographic and clinical characteristics of participants stratified by body roundness index (BRI) tertiles (N = 35,031).

Variables	Total population BRI range 0.686–23.508	BRI first tertile 0.686–4.201	BRI second tertile 4.201–6.054	BRI third tertile 6.054–23.508	*P* value
	(N = 35,031)	(N = 11,683)	(N = 11,683)	(N = 11,665)	
Age (yr)					<.001
≥20, ≤40					
>40, ≤60					
>60					
BRI	5.43 ± 2.35	3.15 ± 0.70	5.09 ± 0.53	8.06 ± 1.87	<.001
PIR	2.50 ± 1.63	2.60 ± 1.68	2.59 ± 1.63	2.32 ± 1.55	<.001
Gender, n (%)					<.001
Male	17,304 (49.40%)	6272 (53.68%)	6407 (54.84%)	4625 (39.65%)	
Female	17,727 (50.60%)	5411 (46.32%)	5276 (45.16%)	7040 (60.35%)	
Race, n (%)					<.001
Mexican American	9050 (25.83%)	2192 (18.76%)	3428 (29.34%)	3430 (29.40%)	
Other Hispanic	14,651 (41.82%)	4952 (42.39%)	4829 (41.33%)	4870 (41.75%)	
Non-Hispanic	7612 (21.73%)	2713 (23.22%)	2201 (18.84%)	2698 (23.13%)	
Other	3718 (10.61%)	1826 (15.63%)	1225 (10.49%)	667 (5.72%)	
Education level, n (%)					<.001
Less than high school	7912 (22.59%)	1841 (15.76%)	2848 (24.38%)	3223 (27.63%)	
High school	7620 (21.75%)	2190 (18.75%)	2633 (22.54%)	2797 (23.98%)	
College or above	17,416 (49.72%)	6228 (53.31%)	5840 (49.99%)	5348 (45.85%)	
Data missing	2083 (5.95%)	1424 (12.19%)	362 (3.10%)	297 (2.55%)	
Marital status, n (%)					<.001
Married/cohabiting	19,869 (59.35%)	5788 (54.63%)	7388 (64.65%)	6693 (58.41%)	
Widowed, divorced, separated, never married	13,594 (40.60%)	4803 (45.33%)	4035 (35.31%)	4756 (41.51%)	
Data missing	17 (0.05%)	4 (0.04%)	4 (0.04%)	9 (0.08%)	
PIR					<.001
≤1.3	10,263 (29.30%)	3331 (28.51%)	3177 (27.19%)	3755 (32.19%)	
1.3 to ≤3.5	12,093 (34.52%)	3830 (32.78%)	4027 (34.47%)	4236 (36.31%)	
>3.5	9753 (27.84%)	3632 (31.09%)	3432 (29.38%)	2689 (23.05%)	
Data missing	2922 (8.34%)	890 (7.62%%)	1047 (8.96%)	985 (8.44%)	
Diabetes mellitus, n (%)					<.001
No	29,455 (84.08%)	11,144 (95.39%)	10,007 (85.65%)	8304 (71.19%)	
Yes	5554 (15.85%)	536 (4.59%)	1668 (14.28%)	3350 (28.72%)	
Data missing	22 (0.06%)	3 (0.03%)	8 (0.07%)	11 (0.09%)	
Hypertension, n (%)					<.001
No	23,197 (66.22%)	9772 (83.64%)	7633 (65.33%)	5792 (49.65%)	
Yes	11,818 (33.74%)	1907 (16.32%)	4044 (34.61%)	5867 (50.30%)	
Data missing	16 (0.05%)	4 (0.03%)	6 (0.05%)	6 (0.05%)	
Depression, n (%)					<.001
No	32,032 (91.44%)	10,904 (93.33%)	10,829 (92.69%)	10,299 (88.29%)	
Yes	2999 (8.56%)	779 (6.67%)	854 (7.31%)	1366 (11.71%)	
Smoking status, n (%)					<.001
Never	18,781 (53.61%)	6248 (53.48%)	6237 (53.39%)	6296 (53.97%)	
Current	8023 (22.90%)	1836 (15.72%)	2977 (25.48%)	3210 (27.52%)	
Former	6924 (19.77%)	2712 (23.21%)	2241 (19.18%)	1971 (16.90%)	
Data missing	1303 (3.72%)	887 (7.59%)	228 (1.95%)	188 (1.61%)	
Alcohol use, n (%)					<.001
Nondrinker	8457 (24.14%)	2411 (20.64%)	2678 (22.92%)	3368 (28.87%)	
Drinker	20,681 (59.04%)	7156 (61.25%)	7229 (61.88%)	6296 (53.97%)	
Data missing	5893 (16.82%)	2116 (18.11%)	1776 (15.20%)	2001 (17.15%)	
Physical activity, n (%)					<.001
Activity	17,617 (50.29%)	5617 (48.08%)	5784 (49.51%)	6216 (53.29%)	
Less activity	2319 (6.62%)	780 (6.68%)	754 (6.45%)	785 (6.73%)	
Inactivity	10,323 (29.47%)	3540 (30.30%)	3521 (30.14%)	3262 (27.96%)	
Data missing	4772 (13.62%)	1746 (14.94%)	1624 (13.90%)	1402 (12.02%)	
Dyslipidemia, n (%)					<.001
No	12,963 (37.00%)	6008 (51.43%)	3675 (31.46%)	3280 (28.12%)	
Yes	20,492 (58.50%)	5046 (43.19%)	7552 (64.64%)	7894 (67.67%)	
Data missing	1576 (4.50%)	629 (5.38%)	456 (3.90%)	491 (4.21%)	
Sleeplessness, n (%)					<.001
No	26,393 (75.34%)	9442 (80.82%)	9007 (77.09%)	7944 (68.10%)	
Yes	8626 (24.62%)	2237 (19.15%)	2671 (22.86%)	3718 (31.87%)	
Data missing	12 (0.03%)	4 (0.03%)	5 (0.04%)	3 (0.03%)	
Cardiovascular diseases, n (%)					<.001
No	29,561 (84.39%)	9723 (83.22%)	10,179 (87.13%)	9659 (82.80%)	
Yes	3407 (9.73%)	542 (4.64%)	1150 (9.84%)	1715 (14.70%)	
Data missing	2063 (5.89%)	1418 (12.14%)	354 (3.03%)	291 (2.49%)	

Data are expressed as mean ± standard deviation, median (interquartile range), or percentage, as appropriate. Missing data for covariates included: education level (2083 (5.95%)), marital status (17 (0.05%)), PIR (2922 (8.34%)), diabetes mellitus (22 (0.06%)), hypertension (16 (0.05%)), smoking status (1303 (3.72%)), alcohol use (5893 (16.82%)), physical activity (4772 (13.62%)), dyslipidemia (1576 (4.50%)), sleeplessness (12 (0.03%)), and cardiovascular diseases (2063 (5.89%)).

BRI = body roundness index, PIR = poverty-to-income ratio.

### 3.2. Unadjusted association between baseline BRI and the risk of depression

Table 2 shows that BRI was significantly associated with depression. Each 1-unit increase in BRI raised the risk by 13% (OR = 1.13, 95% CI: 1.12–1.15, *P* < .0001). Compared to the lowest tertile, the highest tertile showed an 86% higher risk (OR = 1.86, *P* < .0001). Biochemical factors such as dyslipidemia (OR = 1.25, *P* < .0001), diabetes (OR = 1.63, *P* < .0001), and hypertension (OR = 1.71, *P* < .0001) were independently associated with depression. Sleeplessness (OR = 4.76, *P* < .0001) and cardiovascular diseases (OR = 2.25, *P* < .0001) also significantly increased depression risk.

**Table 2:** Univariate analysis of the association between baseline variables and depression.

	Statistics	Odds ratio (95% CI)	*P* value
BRI	5.43 ± 2.35	1.13 (1.12–1.15)	<.0001
BRI tertile			
Low	11,683 (33.35%)	1	
Middle	11,683 (33.35%)	1.10 (1.00–1.22)	.0544
High	11,665 (33.30%)	1.86 (1.69–2.04)	<.0001
Gender			
Male	17,304 (49.40%)	1	
Female	17,727 (50.60%)	1.75 (1.62–1.89)	<.0001
Age	47.50 ± 18.62	1.00 (1.00–1.00)	.4941
Race, n (%)			
Mexican American	9050 (25.83%)	1	
Other Hispanic	14,651 (41.82%)	0.85 (0.78–0.94)	.0007
Non-Hispanic	7612 (21.73%)	0.89 (0.80–0.99)	.0314
Other	3718 (10.61%)	0.62 (0.53–0.72)	<.0001
Education level, n (%)			
Less than high school	7912 (22.59%)	1	
High school	7620 (21.75%)	0.68 (0.62–0.76)	<.0001
College or above	17,416 (49.72%)	0.48 (0.44–0.53)	<.0001
Data missing	2083 (5.95%)	0.51 (0.42–0.61)	<.0001
Marital status, n (%)			
Married/cohabiting	19,869 (59.35%)	1	
Widowed, divorced, separated, never married	13,594 (40.60%)	1.86 (1.72–2.01)	<.0001
Data missing	17 (0.05%)	1.89 (0.43–8.28)	.3979
Smoking status, n (%)			
Never	18,781 (53.61%)	1	
Current	8023 (22.90%)	1.27 (1.15–1.40)	<.0001
Former	6924 (19.77%)	2.76 (2.53–3.02)	<.0001
Data missing	1303 (3.72%)	1.03 (0.82–1.30)	.7887
Alcohol use, n (%)			
Nondrinker	8457 (24.14%)	1	
Drinker	20,681 (59.04%)	1.02 (0.93–1.11)	.7308
Data missing	5893 (16.82%)	0.99 (0.88–1.11)	.8571
Diabetes mellitus, n (%)			
No	29,455 (84.08%)	1	
Yes	5554 (15.85%)	1.63 (1.49–1.78)	<.0001
Data missing	22 (0.06%)	0.56 (0.07–4.14)	.5675
Hypertension, n (%)			
No	23,197 (66.22%)	1	
Yes	11,818 (33.74%)	1.71 (1.59–1.85)	<.0001
Data missing	16 (0.05%)	0.88 (0.12–6.65)	.8993
Physical activity, n (%)			
Activity	17,617 (50.29%)	1	
Less activity	2319 (6.62%)	0.99 (0.86–1.16)	.9442
Inactivity	10,323 (29.47%)	0.90 (0.82–0.98)	.0138
Data missing	4772 (13.62%)	0.65 (0.57–0.74)	<.0001
Dyslipidemia, n (%)			
No	12,963 (37.00%)	1	
Yes	20,492 (58.50%)	1.25 (1.15–1.35)	<.0001
Data missing	1576 (4.50%)	1.18 (0.98–1.42)	.0820
Sleeplessness, n (%)			
No	26,393 (75.34%)	1	
Yes	8626 (24.62%)	4.76 (4.41–5.14)	<.0001
Data missing	12 (0.03%)	1.76 (0.23–13.65)	.5883
Cardiovascular diseases			
No	29,561 (84.39%)	1	
Yes	3407 (9.73%)	2.25 (2.03–2.49)	<.0001
Data missing	2063 (5.89%)	0.87 (0.73–1.04)	.1252
PIR			
≤1.3	10,263 (29.30%)	1	
1.3 to ≤3.5	12,093 (34.52%)	0.51 (0.47–0.56)	<.0001
>3.5	9753 (27.84%)	0.25 (0.22–0.28)	<.0001
Data missing	2922 (8.34%)	0.63 (0.55–0.73)	<.0001

Data are expressed as mean ± standard deviation, median (interquartile range), or percentage, as appropriate.

BRI = body roundness index, CI = confidence interval, PIR = poverty-to-income ratio.

### 3.3. J-shaped association between BRI and depression risk

**Figure 2.**
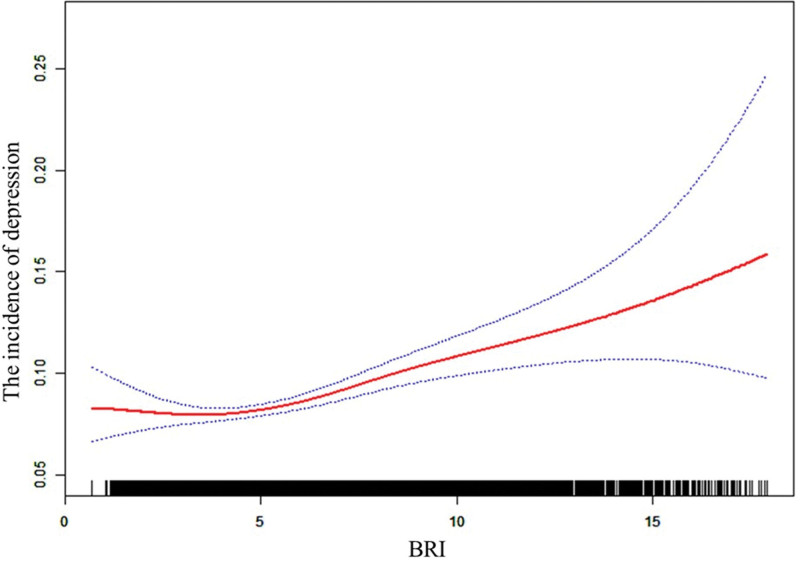
Associations between the BRI and depression in the total population. Associations between the BRI and depression in the total population. A threshold, nonlinear association between BRI and depression was observed using a generalized additive model. The solid red line represents the smooth curve fit between variables, while the blue bands indicate the 95% confidence intervals of the fit. The model was adjusted for age, gender, race/ethnicity, education level, marital status, poverty–income ratio, alcohol consumption, smoking status, hypertension, diabetes, cardiovascular disease, physical activity, and dyslipidemia. BRI = body roundness index.

A J-shaped relationship was observed between BRI and depression risk (Fig. 2). The solid red line represents the adjusted ORs from 2-piecewise linear regression, with the gray band indicates the 95% CI.

This study elucidates a J-shaped relationship between the BRI and the risk of depression, identifying a critical threshold at a BRI value of 3.21 (Table 3). Within the lower BRI range (<3.21), each unit increase in BRI is associated with a 19% reduction in the probability of depression occurrence (OR = 0.81, 95% CI: 0.69–0.96, *P* = .0127). Additionally, an increase of 1 standard deviation in this range corresponds to a significant 39% decrease in depression risk (OR = 0.61, 95% CI: 0.42–0.90, *P* = .0129). In contrast, when the BRI exceeds 3.21, both per-unit and per-standard deviation increases in BRI significantly elevate the risk of depression (per-unit increase OR = 1.06, 95% CI: 1.04–1.08; per-standard deviation increase OR = 1.16, 95% CI: 1.11–1.21, both *P* < .0001). Statistical significance was confirmed by the likelihood ratio test (*P* = .002).

**Table 3:** Threshold effect analysis of the BRI and depression.

Models	Per-unit increase	*P* value	Per-SD increase	*P* value
	OR (95% CI)		OR (95% CI)	
Model I				
One line effect	1.05 (1.03, 1.07)	<.0001	1.13 (1.08–1.17)	
*P* < model II				
Turning point (K)	3.21		−0.94	
BRI < K	0.81 (0.69, 0.96)	.0127	0.61 (0.42–0.90)	.0129
BRI ≥ K	1.06 (1.04, 1.08)	<.0001	1.16 (1.11–1.21)	<.0001
*P* value* for LRT test		.002		.002

Data are presented as OR with 95% CI and *P* values. Model I represents a linear analysis, while model II represents a nonlinear analysis. All models are adjusted for age, gender, race/ethnicity, education level, marital status, poverty–income ratio, alcohol consumption, smoking status, hypertension, diabetes, cardiovascular disease, physical activity, and dyslipidemia.

CI = confidence interval, LRT = likelihood ratio test, OR = odds ratio, SD = standard deviation.

* *P* < .05 indicates that model II significantly differs from model I.

## 4. Discussion

In this cross-sectional study, we analyzed the association between the BRI and depression using data from a nationally representative sample. The results showed a J-shaped relationship, where both lower and higher BRI levels were associated with an increased risk of depression, while individuals with moderate BRI levels had the lowest risk. This relationship remained consistent after adjusting for potential confounders.

Numerous studies have demonstrated a bidirectional relationship between obesity and depression. For instance, Vogelzangs et al using data from the PREVEND cohort in the Netherlands, found that obesity (measured by BMI) was significantly associated with depressive symptoms, particularly among women.^[16]^ However, BMI has been shown to have limitations in its capacity to differentiate between subcutaneous and visceral fat.^[17,18]^ In contrast, the BRI has been demonstrated to offer a more precise reflection of visceral fat distribution.^[19,20]^ This offers a more nuanced understanding of the relationship between obesity and depression risk, expanding on findings from studies like Vogelzangs et al.^[16]^ Similarly, Zhang et al reported a positive association between BRI and depressive symptoms in a large-scale population. Participants in the highest BRI quartile had a significantly elevated risk of depression.^[21]^ While their study primarily focused on a linear relationship, our findings extend their work by identifying a J-shaped association, where both low and high BRI levels are independently associated with increased depression risk.^[21]^ This observation contributes to the current understanding of the relationship between BRI and depression, underscoring the need to consider nonlinear effects in future research.

Our findings are consistent with those of systematic reviews such as that by Swainson et al^[22-24]^ which identified potential mechanisms linking obesity and depression, including chronic inflammation, oxidative stress, and gut microbiota dysregulation. Importantly, our study provides additional insights by demonstrating that low BRI levels, which may reflect malnutrition or insufficient energy reserves, could also increase the risk of depression. The dual risk observed at both ends of the BRI spectrum underscores the importance of balanced body composition for mental health. This finding offers a novel perspective on the complex relationship between adiposity and depression, emphasizing the need for future research to explore these associations further.

The association between BRI and depression can be explained through several biological and psychosocial mechanisms, including chronic inflammation.^[25]^ Elevated BRI reflects increased visceral fat, which in turn releases pro-inflammatory cytokines such as TNF-α, IL-6, and CRP. These inflammatory markers can cross the blood–brain barrier, disrupting central nervous system function and impairing neurotransmitter regulation, particularly serotonin. Furthermore, inflammation can lead to overactivation of the hypothalamic–pituitary–adrenal axis, a pathway that is closely associated with the development of depression. Another significant mechanism involves the gut–brain axis, where elevated BRI is with alterations in gut microbiota composition, including an increase in potentially harmful bacteria. These changes can compromise gut barrier function, leading to systemic inflammation, while microbial metabolites (e.g., short-chain fatty acids) can negatively affect brain function via vagal or inflammatory pathways,^[26]^ Other mechanisms,^[27]^ including oxidative stress,^[27]^ hormonal dysregulation,^[28]^ and psychosocial factors may also play significant roles.^[29,30]^

This study possesses notable strengths that enhance the reliability of its findings. It comprehensively adjusted for confounding factors, including demographics, lifestyle behaviors, and comorbidities, minimizing bias and strengthening the validity of the associations. By utilizing BRI as the exposure variable, which accounts for body shape and fat distribution, the study provides a detailed assessment of body composition’s link to depression. Additionally, advanced statistical methods, such as threshold effect analysis and GAM, were employed to reveal the nonlinear J-shaped relationship between BRI and depression.

This study has certain limitations. First, individuals under the age of 20 were excluded from the study. Second, as a cross-sectional study, this research cannot establish a causal relationship between BRI and depression, and longitudinal studies are required for further validation. Third, the predictive ability of BRI for depression may vary across different populations, such as by ethnicity, sex, or BMI category, and further investigations in broader and more diverse populations are necessary. Finally, although multiple confounding factors were adjusted for, the possibility of unmeasured factors (e.g., dietary patterns or life stress) influencing the association between BRI and depression cannot be ruled out.

## 5. Conclusion

This study employed data from the NHANES database (2005–2018) to examine the relationship between BRI and depression in adults aged 20 years and older. The analysis encompassed a total of 35,031 participants. The findings revealed a nonlinear dose–response relationship between BRI and the prevalence of depression. The association followed a J-shaped curve, with both higher and lower BRI levels correlating with an elevated risk of depression.

## Acknowledgments

We thank to XL Chen (Yi-er college) for her work on the NHANES database. We thank XL Chen for her outstanding work and invaluable encouragement, which made it easier for us to explore this area of research.

## Author contributions

**Conceptualization:** Jian Li.

**Data curation:** Jian Li.

**Formal analysis:** Jian Li.

**Software:** Jian Li.

**Validation:** Lichun Zhou.
